# Adverse childhood experience and adult persistent pain and disability: protocol for a systematic review and meta-analysis

**DOI:** 10.1186/s13643-020-01474-8

**Published:** 2020-09-17

**Authors:** André Bussières, Jan Hartvigsen, Manuela L. Ferreira, Paulo H. Ferreira, Mark J. Hancock, Laura S. Stone, Timothy H. Wideman, Jill Boruff, Ask Elklit

**Affiliations:** 1 School of Physical and Occupational Therapy, Faculty of Medicine, McGill University, 3630 Promenade Sir-William-Osler, Montreal, Quebec, H3G 1Y5 Canada; 2 Département Chiropratique, Université du Québec à Trois-Rivières, 3351, boul. Des Forges, C. P. 500, Trois-Rivières, Québec, G9A 5H7 Canada; 3 Department of Sports Science and Clinical Biomechanics, University of Southern Denmark, Campusvej 55, DK-5230 Odense M, Denmark; 4 Nordic Institute of Chiropractic and Clinical Biomechanics, Campusvej 55, DK-5230 Odense M, Denmark; 5 Institute of Bone and Joint Research, The Kolling Institute, Northern Clinical School, Faculty of Medicine and Health, The University of Sydney, Level 10, Kolling Building, Royal North Shore Hospital, St Leonards, Sydney, NSW 2065 Australia; 6 Musculoskeletal Health, Faculty of Health Sciences, Discipline of Physiotherapy, The University of Sydney, Room 155, O Block, Cumberland Campus C42, Sydney, NSW 1825 Australia; 7 Faculty of Medicine and Health Sciences, Macquarie University, Balaclava Road, North Ryde, Sydney, NSW 2109 Australia; 8 Faculty of Dentistry, McGill University, 2001 Av McGill College #500, Montreal, Quebec, H3A1G1 Canada; 9 Alan Edwards Centre for Research on Pain, McGill University, 845 Sherbrooke Ouest, Montreal, Quebec, H3A 0G4 Canada; 10 Faculty of Medicine, Department of Anesthesiology, 420 Delaware Street SE MMC 294, Minneapolis, MN 55455 USA; 11 Schulich Library of Physical Sciences, Life Sciences, and Engineering, McGill University, Macdonald-Stewart Library Building, 809 Sherbrooke Street West, Montreal, Quebec, H3A 0C1 Canada; 12 National Centre for Psychotraumatology, Department of Psychology, University of Southern Denmark, Campusvej 55, DK-5230 Odense M, Denmark

**Keywords:** Adverse childhood experiences, Emotional abuse, Physical abuse, Sexual abuse, Neglect, Persistent pain, Musculoskeletal pain, Somatic pain, Systematic review, Meta-analysis

## Abstract

**Background:**

A growing body of research highlights the pervasive harms of adverse childhood experiences (ACEs) on health throughout the life-course. However, findings from prior reviews and recent longitudinal studies investigating the association between types of ACEs and persistent pain have yielded inconsistent findings in the strength and direction of associations. The purpose of this review is to appraise and summarize evidence on the relationship between ACEs and persistent pain and disability outcomes in adulthood. The specific aims are (1) to determine whether there is a relationship between exposure to ACE and persistent pain and disability in adults and (2) to determine whether unique and cumulative ACEs exposures (number and type) increase the risk of developing persistent pain and disability in adulthood.

**Method:**

A systematic review and meta-analysis of observational studies will be conducted. Our eligibility criteria are defined following a PECOS approach: *population,* adults with persistent (≥ 3 months) musculoskeletal and somatoform painful disorders *exposed* to single or cumulative direct ACEs alone (i.e., physical, sexual, emotional abuse or neglect) or in combination to indirect types of ACE (e.g., parental death, exposure to domestic violence) in the first 18 years of life; *comparators,* unexposed individuals; *outcomes,* measurements for persistent pain (≥ 3 months) and disability using discrete and/or continuous measures; and *settings,* general population, primary care. A comprehensive search of MEDLINE (Ovid) and nine other pertinent databases was conducted from inception to 29 August 2019 using a combination of key words and MeSh terms (the search will be updated prior to conducting the analyses). Pairs of reviewers will independently screen records and full text articles, and a third reviewer will be consulted in cases of disagreement. Data will be extracted using Endnote and Covidence and a meta-analysis will be conducted using Review Manager (RevMan) Version 5.3. The Scottish Intercollegiate Guidelines Network (SIGN) and the Joanna Briggs Institute (JBI) checklists will be used to assess the quality of the included studies. If heterogeneity is high, the findings will be presented in narrative form.

**Discussion:**

The present review will help consolidate knowledge on persistent pain and disability by evaluating whether frequency and type of adverse childhood experiences produces the most harm. Findings may help inform practitioners and policy-makers who endeavor to prevent and/or mitigate the consequences of ACEs and promote healthy development and well-being of children, youth, and families.

**Systematic review registration:**

PROSPERO CRD42020150230

## Background

Adverse childhood experiences (ACEs) include harms that affect children or adolescence directly (e.g., physical, sexual or emotional abuse or neglect) by a parent, caregiver, or another person in a custodial role (e.g., clergy, coach, teacher) [1, 2] and indirectly through their living environments (e.g., parental conflict, substance or alcohol abuse, mental illness, death or severe sickness of a parent or sibling) [3, 4]. While a growing body of research highlights the pervasive harms of ACEs on health throughout the life-course [5–7], and ultimately premature mortality [3], the role of ACEs in development of persistent pain and disability in adulthood remains controversial.

Prior reviews have revealed the long-term effects of ACEs on developmental disruptions, negative adult psychological and physical health outcomes, risky health behaviors, increased healthcare utilization [5, 8–12], and associated high societal financial burden [13, 14]. The total annual costs attributable to ACEs for six causes of ill health (cancer, diabetes, cardiovascular disease, respiratory disease, anxiety, depression) is estimated to range between US$417 and $487 billion in Europe and North America, with over 75% of the cost arising in individuals with two or more ACEs [12].

It is estimated that a billion children aged 2–17 years were victims of violence worldwide in 2014 [15]. The global prevalence of self-reported child physical and sexual abuse are estimated at 22.6% [16] and 11.8% [17], respectively. The combined global prevalence of self-reported child physical and emotional neglect is estimated at 17% [18, 19].

There is increasing evidence that ACEs are associated with persistent pain [20–24] and disability [12] in adulthood. A 30-year prospective follow-up of a cohort of individuals with court documented early ACEs and a demographically matched control sample showed a small (partial eta squared (*η*^2^) = .01), but significant increase in risk of pain symptoms in middle adulthood [25]. In this cohort, a history of ACEs and the presence of post-traumatic stress disorders (PTSD) in adulthood led to a significant increase in most types of pain complaints, where PTSD alone did not. A 2009 meta-analysis found that childhood sexual abuse (CSA) was associated with a complaint of musculoskeletal (MSK) or general pain (Cohen’s *d* = 0,39, 95% CI 0.15–0.61) [26]. Self-reported physical abuse in childhood also appears to increase the risk for reporting back and neck pain [27–30], but not sexual abuse [27]. Those experiencing a greater number of ACEs are at increased risk of adult-onset neck/back pain (1–2ACEs: *adj* hazard ratio 1.41, 95% CI 1.04–1.93 vs ≥ 3ACEs: 2.86, 1.66–4.94) compared to those with no ACE [29]. Data on the relationship between self-reported ACEs and adult-onset arthritis is less clear however [10, 31–34].

Self-reported ACEs have also been associated with adult widespread pain and fibromyalgia [35, 36], migraine [37, 38] and frequent headache [39], irritable bowel syndrome [40], non-cardiac chest pain [26, 41], and chronic pelvic pain [23, 26, 42–45]. In contrast, a 2009 review did not find a significant association between childhood or adulthood sexual abuse and a lifetime diagnosis of fibromyalgia [45].

Many of the above studies had small sample size and were retrospective, and self-reported data based on interviews and questionnaires is subject to recall bias. While some reviews strongly support a relationship between AECs and persistent pain [46–48], others suggest that the relationship is modest at best [10, 23, 26, 36, 49, 50], or that the evidence is limited [51]. Cross-sectional studies generally report an association for various forms of persistent pain, but such studies are prone to quality-of-evidence problems, and do not provide information on incidence. Some longitudinal studies evaluating the abuse-pain relationship support an association with MSK pain [27, 28, 52, 53], others do not [54, 55]. Recent reviews suggests an increased risks of poor health outcomes with multiple ACE exposures (≥ 2 vs no ACE [12] and ≥ 4 vs no ACE [5]). It remains unknown however if a dose-response relationship also exists for persistent pain and disability. To our knowledge, there has been no attempt to summarize available evidence about the effect of cumulative ACEs (number and type) as risk factors for adult persistent pain and disability. In addition, the quantity and quality of research linking ACEs with somatic pain is sparse, research is generally limited to small, convenience samples, with insufficient attention to the design of control groups and to sample size [56].

### Objectives

The overall purpose of this review is to appraise and summarize evidence on the relationship between adverse childhood experiences and persistent pain (MSK and somatoform pain disorders) and disability outcomes in adulthood.

The specific aims are as follows:

1. To determine whether there is a relationship between exposure to ACE and persistent pain and disability in adults
2. To determine whether unique and cumulative ACEs exposures (number and type) increase the risk of developing persistent pain and disability in adulthood

## Methods/design

The following protocol has been written according to the MOOSE Guidelines for Meta-Analyses and Systematic Reviews of Observational Studies and the PRISMA-P (Preferred Reporting Items for Systematic Reviews and Meta-Analyses Protocols) guidelines [57, 58]. The completed checklist can be found as an Additional file 1 to this document. Further, we considered recent recommendations on methods to systematically review and meta-analyses of observational studies when developing this protocol [59, 60]. The protocol has been registered at the International Prospective Register of Systematic Reviews (PROSPERO; registration number CRD42020150230).

### Eligibility criteria

To identify relevant studies, specific inclusion and exclusion criteria have been identified using the population, exposure, controls, outcomes, setting, and study designs (PECOS) criteria as follows:

#### Population

Adults (age 18 years or over) with persistent (≥ 3 months) musculoskeletal or somatoform painful disorders. For the purpose of this review, persistent pain is defined as pain that has lasted for more than 3 months.

#### Exposure

Adverse childhood experience. Terms such as childhood maltreatment [61], childhood trauma [62], stressful experiences in childhood [28], early-life adversity [63], childhood adversities, and childhood psychosocial stressors [33] have been used interchangeably to describe childhood events, that are varying in severity and often chronic, occurring within a child’s family or social environment that cause harm or distress, thereby disrupting the child’s physical or psychological health and development [64]. This review will use the term adverse childhood experiences (ACEs) which links either directly to four main types of childhood trauma (physical, sexual, emotional abuse, and neglect) or in combination to indirect types of ACEs, while emphasizing a core psychological aspect of harm. In the current protocol, direct types of ACEs are defined according to the World Health Organization and International Society for Prevention of Child Abuse and Neglect [2]:

1. Childhood physical abuse (CPA) is defined as the intentional use of physical force against a child that results in (or has a high likelihood of resulting in) harm to the child’s health, survival, development, or dignity. This included hitting, beating, kicking, shaking, biting, strangling, scalding, burning, poisoning, and suffocating the child.
2. Childhood sexual abuse (CSA) is defined as the involvement in sexual activity that a child does not fully comprehend, is unable to give informed consent to, or for which the child is not developmentally prepared [2] or else that violates the laws or social taboos of society. Children can be sexually abused by both adults and other children who are—by virtue of their age or stage of development—in a position of responsibility, trust, or power over the victim.
3. Childhood emotional abuse (CEA) involved both isolated incidents as well as a pattern of failure over time on the part of the parent or caregiver to provide a developmentally appropriate and supportive environment to the child. This included the restriction of movement, patterns of belittling, blaming, threatening, frightening, discriminating against, or ridiculing and other non-physical forms of rejection or hostile treatment.
4. Childhood neglect (CN) included both isolated incidents as well as a pattern of failure over time on the part of a parent or caregiver to provide for the development and well-being of the child (where the parent is in a position to do so) in one or more of the following areas: health, education, emotional development, nutrition, shelter, and safe living conditions.

In this review, harms that affect children indirectly through their living environments (i.e., indirect types of ACE) include the following terms [5, 65]:

- Exposure to domestic violence, family violence, frequent fear of family member, bullying, victim or witness of violent crime, witness of neighborhood violence or community violence, war, or disaster
- Parental divorce/separation, absence of a parental figure, or single-parent family
- Family conflict/discord, household dysfunction, family instability, poor parent-child relationship, low parental education, or non-intact family
- Separation from family, out-of-home care, child protection record, child custody, child care, or foster care
- Parental/caregiver death or death of a close relative or friend
- Discrimination
- Serious childhood illness/injury/accident or severe family illness
- Poverty, economic hardship, family financial problems, economic adversity, childhood hunger, low standard of living, low socioeconomic status, or parental unemployment
- Household mental illness, parental mental disorder, or household alcohol and drug abuse
- Household criminality/jailed/imprisoned, or parental criminal behavior or involvement in juvenile justice

#### Comparators

Individuals who did not report experiencing ACE in childhood

#### Outcome measures

Presence of persistent symptoms (lasting ≥ 3 months) and disability (if available), along with measurement time points will be extracted and reported. Where possible, a meta-analysis will be performed to estimate the effect of ACEs on persistent pain and disability.

Secondary outcomes of interest include the following:

- How does “number” of ACEs affect persistent pain and disability outcomes?
- How does “type(s)” of ACEs affect persistent pain and disability outcomes?
- Are there potential gender differences in persistent pain and disability consequences with regard to ACEs?
- Where available, will such an association withstand the control of age, sex, education, socioeconomic status, and early mental disorders (e.g., depression and anxiety )[21]?

#### Setting

General population, primary care

#### Designs

Observational studies including cross-sectional, case control, nested case–control, or cohort (retrospective or prospective, longitudinal, and population-based studies).

### Including criteria

For inclusion, studies need to meet the following criteria:

1. Report single or cumulative measures of ACE exposures in the childhood period (first 18 years of life) spanning either direct types (childhood sexual, physical, emotional abuse or neglect) alone or in combination to indirect types (e.g., household dysfunction, parental death, family violence) and compare them with individuals who did not suffer ACE in childhood
2. Report definitions and measurements for persistent MSK or somatoform pain (≥ 3 months) and disability (if available) using discrete (e.g., relative risk (RR), odds ratio (OR), hazard ratio (HR) or incidence rate ration (IRR)) and/or continuous outcome measures (e.g., mean difference (MD), standardized mean difference (SMD), Cohens *f*, Hedges’ *g*) for one ACE and multiple ACEs; MSK pain disorders include non-specific spine pain (neck, back, low back pain), upper extremity (shoulder, elbow, wrist, hand), and lower extremity pain (hip, knee, ankle or foot). Somatoform painful disorders include conditions such as widespread pain, fibromyalgia, frequent headache, migraine, abdominal, non-cardiac, and gynecological pain syndromes. Disability includes indicators of pain-related disability such as activity limitation
3. Test the associations between (1) and (2)
4. Be an observational study examining the link between ACEs and persistent pain and disability (if available) in adulthood with a sample size of at least 100 in each group [5] or 200 for continuous outcomes

#### Language of publication

Considering the languages spoken by co-authors, studies conducted in any country and reported in English, French, Portuguese, Spanish, and any Scandinavian language are eligible for inclusion.

### Exclusion criteria

We will exclude studies based on high-risk (e.g., the homeless, those in prison or individuals with a primary diagnosis of a substance use disorder) populations because there are often few individuals with low ACE exposure in such populations [5]. The following studies will also be excluded: studies investigating the assessment or the treatment of persistent pain and related conditions or mechanisms underlying persistent pain and studies focused mainly on individuals younger than 18 years or on adults with pain that have a clear or predominant tissue injury component (e.g., nociceptive pain from bone fractures, sprains or burns, neuropathic pain, cancer-related pain). Single case studies and randomized controlled trials will be excluded. When findings from iterations of the same survey were reported, we will include data only from the most recent survey.

### Information sources

Academic databases are as follows: MEDLINE (Ovid), Embase (Ovid), PsycInfo (Ovid), Social Work Abstracts (Ovid), CINAHL (EBSCO), SocIndex (EBSCO), Sociological Abstracts (includes Social Services Abstracts) (ProQuest) and International Bibliography of Social Sciences (ProQuest), Dissertations and Theses (ProQuest), and Web of Science Core Collection. We used search filters to identify observational studies in Medline, Embase, and CINAHL [66].

### Search strategy

A health sciences librarian developed the search strategy and performed the literature searches in the abovementioned ten databases from database inception until August 29, 2019, with no language restrictions and with the observational studies limit as mentioned above (the search will be updated prior to conducting the analyses). The MEDLINE strategy was developed with input from the project team and peer-reviewed by a second librarian using the PRESS standard [67]. After the initial MEDLINE strategy was finalized, it was adapted for use in the other databases. The search strategy [see Additional file 2] was designed to identify all relevant clinical literature reporting associations and risks of persistent pain from single or multiple ACEs. Bibliographies of relevant articles were hand-searched for other relevant articles.

### Team expertise

Team expertise relevant for the subject matter is as follow: AB has methodological expertise in conducting systematic review related to MSK pain, JH has expertise in conducting longitudinal studies, MLF has expertise in the cause and prognosis of back pain, PHF and MJH have expertise in the diagnosis and management of MSK pain, LSS has expertise in the biological mechanisms of MSK pain, THW investigates the biopsychosocial risk factors for persistent pain-related disability, JB is a health sciences librarian, and AE is an expert in psychotraumatology. JH, MLF, PHF, and MJH have expertise in conducting reviews dealing with MSK pain.

### Data management

To organize the complete search result from the 10 different databases, the candidate studies were imported from their respective databases to Endnote and duplicates removed. These duplicates will be quarantined. From there, the references will be imported into Covidence.

### Selection process

We will use a 2-step process to assess the results of the literature search (screening and full text review). Pairs of reviewers will independently screen citations and abstracts based on the inclusion criteria using a standardized screening sheet. The first and second author will first double screen 25% of the references in order to establish coder reliability at this stage. If the Cohen’s kappa inter-rater reliability for inclusion or exclusion, as indicated by Cohen’s kappa, is satisfactory (above .80), the remaining references will screened independently by the two coders. If the inter-rater reliability is below .80 the two screeners will go through their conflicts and agree on the criteria before continuing screening. Any disagreements will be resolved through discussions and by consulting the original paper. If the abstracts do not provide sufficient information to determine inclusion or exclusion (i.e., “can’t tell” on the aforementioned questions), the reference will be included in the next stage (full text screening) in order to confer with information given in the full text. Once the full texts are available, team members will proceed similarly. Any discrepancies will be discussed and resolved through consensus or a third reviewer if needed.

### Data collection process

A calibration exercise using the first 10–15 consecutive studies will be undertaken to pilot test and refine our data extraction form adapted from Jadhakhan et al. [68] [see Additional file 3]. For each study, pairs of reviewers will independently extract data and reach consensus through discussion. A third reviewer will be used to resolve disagreements if consensus cannot be reached. Extracted information will include general study characteristics (title and first author, year of study, geographical location); study characteristics: study design and method of data-analysis; participants: study population, number of participants in each group, and patient characteristics such as age, gender, and co-morbidities; AEC exposure (type, description, setting, frequency of abuse, self-report vs objectively verifiable methods); outcome measurement (pain type, disability and timeframe), diagnosis of post-traumatic stress disorder, and psychological and mental disorder; comparison group; and impact of exposure on persistent MSK or somatoform painful conditions. Following precedent in the literature [3, 5, 10], unadjusted and adjusted estimates for discrete (RRs, ORs, HRs, IRRs) and continuous (MDs, SMDs, *F* test results) outcomes and measures of precision (confidence intervals, *P* values, standard deviations, standard errors) will be extracted at each available ACE count level (for demographics and socioeconomic status where available) versus those with none. When multiple studies reported data for the same sample and outcome, we will include one study on the basis of largest sample size or with the longest follow-up time (closest fit to study requirements). Where several study outcomes can be contributed to a single persistent pain condition, two reviewers will independently identify the best match, with a third reviewer resolving any conflicts. For studies with insufficient data to evaluate the eligibility, we will contact the study authors by email at least twice for clarification. The study will be excluded if there is still insufficient data following this process.

### Risk of bias assessment

Included articles will be independently assessed for methodological quality by pairs of assessors using a modified form adapted from the Scottish Intercollegiate Guidelines Network (SIGN) checklist to assess the quality of the cohort and case–control [69], and the Joanna Briggs Institute (JBI) tool for cross-sectional studies [70]. A narrative summary of the quality for each study will be provided in a table. Any discrepancies will be resolved through discussion with a third reviewer. A critical appraisal of the study quality describing the impact of the quality of each study on the results will be discussed. A sensitivity analysis will be conducted to assess the effect of including or excluding poor quality studies on the main findings.

### Planned methods of analysis

#### Description of studies

We will perform a qualitative synthesis of findings and stratified results based on the type of persistent pain disorders (MSK or somatoform) and direct (sexual abuse, physical abuse, emotional abuse, and neglect) and indirect AEC exposures (e.g., death of parent or close relative). Results from the studies will be summarized and tabulated according to the variables listed above and discuss data in a narrative review. Where statistical pooling is not possible, the findings will be presented in narrative form.

#### Quantitative synthesis

The primary outcomes of interest will be pooled measures of relations between unique or cumulative measures of ACE exposures and persistent pain and disability in adulthood, with individuals reporting no ACEs as the comparator.

Articles reporting some sociodemographic adjustments and adjusted ORs will be transformed into RRs [71]. Following precedent in the literature [12]. Adjusted positive and negative counts by condition and ACE category will be generated for use in the meta-analysis. Covariates from proportional hazards models will be treated as RRs. For each study, all categories of RRs for more than one ACE will be combined to give an RR for two or more ACEs by use of a weighted mean method with weighting by proportions in each ACE category. Where there was only one study, the RR will be calculated directly from that study. The standardized mean difference (Cohen’s *d*) effect size statistic will be used when comparing two no experimentally defined groups on a continuous outcome not uniformly operationalized across studies.

Where appropriate, we will calculate pooled estimates for crude and adjusted RRs or Cohen’s *d* with 95% CIs separately for the risk of persistent pain and disability outcomes among individuals exposed to at least two types of AEC (vs no ACE) in RevMan V.5.3 (Review Manager (RevMan) Version 5.3 [program]. *P* values less than 0.05 will be considered significant. When RRs are presented at a subgroup level within samples, we will pool RRs before analysis. Studies investigating the relationship between reports of ACEs and persistent pain are heterogeneous in regard to conceptualization, sampling, design, and so forth. Therefore, we will perform a random-effects model to investigate the size of the association between types of ACE and persistent pain disorders [72]. We will calculate pooled RRs with the DerSimonian and Laird method and pooled prevalence of individuals with one ACE and with two or more ACEs with the Stuart–Ord method [12].

#### Investigation of heterogeneity and inconsistency

We will use visual inspection of forest plots and the *I*^2^ statistic to assess heterogeneity among pooled studies. Prespecified subgroup analyses will be conducted to explore consistency across results by various factors including study design (cohort design vs case–control design), type of condition (MSK pain vs somatic pain), and if there is at least 2 studies available [36]. To test the hypotheses of a subgroup effect, a test of interaction with a predetermined 2-tailed alpha level of 0.05 will be used [73]. We will conduct sensitivity analyses by excluding outlying studies (study 95% CIs do not overlap those of pooled measures) and by exploring the impact of the risk of different biases on the results in regression analyses.

When possible we will also explore potential sources of heterogeneity, for outcomes with at least ten samples and high heterogeneity between estimates, by meta-regression. We will do univariate analyses using Stata version 12 (Stata Statistical Software: Release 12 [program]. College Station, TX: StataCorp LP, 2011) to test the individual association of the following covariates (when relevant) with pooled estimates: number of ACEs measured (fewer than 4 vs 4 or more), outcome timeframe (recent vs lifetime), and adjusted vs unadjusted estimates.

#### Publication bias

We will explore risk of publication bias using visual inspection of funnel plots (plots of effect estimates against its standard error) when sufficient studies (at least ten samples) can be included in the meta-analysis. Publication bias may lead to asymmetrical funnel plots [74]. We will generate forest plots showing RRs and/or Cohens’ *d* and 95% CIs for each study and the overall random-effects pooled estimate. We will also perform the Begg and Mazumdar rank correlation test and Egger tests [36].

### Dissemination

The findings of this review will be submitted for publication in a peer-reviewed journal.

## Discussion

Prior reviews have highlighted the negative impact of ACEs on psychological (anxiety, depression, self-harm) [6], behavioral (sexual risk taking, smoking, problematic alcohol and drug use, violence), and physical health (overweight or obesity, diabetes, cancer, heart and respiratory disease) [5]. However, the impact of ACE on persistent pain and disability is less clear. A better understanding of these relationships has important public health implications. Because persistent MSK and somatoform painful disorders are very common, even a small increase in risk implies a large number of additional cases and a significant impact on population health.

We expect to encounter several limitations while conducting the systematic review. The varying terminology and definitions of ACEs may make the search strategy less sensitive and results difficult to compare. The review will aim to overcome these limitations by having two information specialists overlook the search strategy and by performing relevant subgroup analyses. This does apply not only to ACEs, but also to MSK and somatoform painful disorders which are clinical diagnosis with little or no objective findings.

Findings from this review may help inform practitioners and policy-makers who endeavor to prevent and/or mitigate the consequences of ACEs and promote healthy development and well-being among children, youth, and families. Conducting a robust synthesis of the evidence is particularly important in light of the increasing global burden of persistent pain [75, 76], and musculoskeletal pain in particular [77–79], our limited understanding of the pathogenesis of persistent pain and underlying vulnerabilities [80], the marginal effectiveness and poorly documented cost-effectiveness for most evidence-based treatments for these conditions [81, 82], the uncertainty about reliability of conclusions regarding prognostic factors for MSK pain [83, 84], and the unexplained variance between individuals (about 55%) due to unknown or unmeasured factors [85].

This review will lay the ground for innovative empirical studies aiming to better understand predictors of MSK and somatoform pain disorders, the complex underpinning biological mechanisms, the implementation of feasible and valid measurement methods in practice [65], and designing and development and testing of effective measures aimed at reducing the impact of ACEs later in life.

## Supplementary information

**Additional file 1:** PRISMA-P 2015 Checklist.

**Additional file 2:** Search strategy in Medline.

**Additional file 3:** Data extraction tables.

## Data Availability

All data and materials used at this step are available in our protocol. All data and materials used during the review will be available from the corresponding author.

## Abbreviations

ACE: Adverse childhood experience
CPA: Childhood physical abuse
CSA: Childhood sexual abuse
CEA: Childhood emotional abuse
CN: Childhood neglect
JBI: Joanna Briggs Institute
MSK: Musculoskeletal
PRISMA-P: Preferred Reporting Items for Systematic Reviews and Meta-Analyses Protocols
PTSD: Post-traumatic stress disorder
SIGN: Scottish Intercollegiate Guidelines Network
