# Supplementary material for: Adverse childhood experience and adult persistent pain and disability: protocol for a systematic review and meta-analysis
Source: Syst Rev. 2020 Sep 17;9:215. doi: 10.1186/s13643-020-01474-8 (PMC7495859; doi:10.1186/s13643-020-01474-8)
Supplement: Supplementary file 2 — Additional file 2: Search strategy in Medline. [file 13643_2020_1474_MOESM2_ESM.docx]

**Additional file 2:** Search strategy for Ovid MEDLINE(R) ALL 1946 to August 29, 2019

1 Chronic Pain/

2 (chronic adj2 pain).tw,kf.

3 Migraine Disorders/

4 Headache/

5 Abdominal Pain/

6 Vulvodynia/

7 Vaginismus/

8 back pain/ or low back pain/ or neck pain/ or Musculoskeletal Pain/

9 Fibromyalgia/ or Temporomandibular Joint Disorders/ or irritable bowl syndrome/

10 exp connective tissue disease/ or arthralgia/ or shoulder pain/ or arthritis/ or exp osteoarthritis/

11 (exp lower extremity/ or exp upper extremity/) and *pain/

12 (somatoform pain or complex pain or functional pain or non-organic pain or somatic pain or psychiatric pain or idiopathic pain or irritable bowel syndrome or genitourinary pain or pelvic pain or orofacial pain or temporomandibular pain or Migraine or abdominal pain or cardiac pain or gynecological pain or Vulvodynia or vaginismus or Headache or bodily pain or back pain or neck pain or widespread pain or fibromyalgia or spine pain or upper extremity pain or shoulder pain or elbow pain or wrist pain or hand pain or hip pain or knee pain or ankle pain or foot pain or lower extremity pain or osteoarthritis or arthritis).tw,kf.

13 1 or 2 or 3 or 4 or 5 or 6 or 7 or 8 or 9 or 10 or 11 or 12

14 violence/ or domestic violence/ or elder abuse/ or spouse abuse/ or intimate partner violence/

15 crime/ or homicide/ or sex offenses/

16 exp warfare/ or accidents/ or disasters/ or natural disasters/

17 bullying/

18 Stress Disorders, Post-Traumatic/ or Stress, psychological/

19 exp socioeconomic factors/

20 poverty.tw,kf.

21 Anxiety/

22 ((exposure or witness or victim) adj3 (crime* or war or disaster* or accident* or violence or abuse or neglect or bully*)).ti,ab,kf.

23 or/14-22

24 (early life or teen or teenage* or child or children or childhood or adolescen*).tw,kf.

25 23 and 24

26 substance-related disorders/ or alcohol-related disorders/ or alcoholism/ or drug overdose/

27 suicide/ or suicide, attempted/ or depressive disorder/ or depression/ or anxiety disorders/

28 Unemployment/

29 Divorce/

30 or/26-29

31 (family or parent* or household or mother or father or foster home* or foster care or childcare or child care or child custody).ti,ab,kf.

32 30 and 31

33 family conflict/ or maternal deprivation/ or paternal deprivation/ or family separation/ or single-parent family/

34 parental death/ or maternal death/

35 "Adult Survivors of Child Abuse"/

36 exp child abuse/

37 adverse childhood experiences/

38 ((early life or teen or teenage* or Child or children or childhood or adolescen*) adj3 (adversity or stressor* or stress or stressful or abuse or abused or trauma or neglect* or adverse or maltreatment or illness or injury or hunger or assault or rape or anxiety or distress)).tw,kf.

39 ((Parent* or mother or father or Family or household or foster home* or foster care or childcare or child care or child custody) adj2 (divorce* or discord or conflict or dysfunction* or instability or separation or mental illness* or mental disorder* or anxiety or depression or substance abuse or drug abuse or alcoholic or alcohol abuse or criminality or criminal or incarcerated or incarceration or unemployment or financial or education or severe illness or serious illness or suicide)).tw,kf.

40 (Death adj2 (parent* or mother or father or family or friend or relative)).tw,kf.

41 25 or 32 or 33 or 34 or 35 or 36 or 37 or 38 or 39 or 40

42 13 and 41

43 Epidemiologic Studies/

44 exp case control studies/

45 exp cohort studies/

46 case control.tw,kf.

47 (cohort adj (study or studies)).tw,kf.

48 cohort analy*.tw,kf.

49 (follow up adj (study or studies)).tw,kf.

50 (observational adj (study or studies)).tw,kf.

51 longitudinal.tw,kf.

52 retrospective.tw,kf.

53 cross-sectional.tw,kf.

54 cross-sectional studies/

55 Incidence/

56 or/43-55

57 42 and 56
