# Supplementary material for: Adverse childhood experience and adult persistent pain and disability: protocol for a systematic review and meta-analysis
Source: Syst Rev. 2020 Sep 17;9:215. doi: 10.1186/s13643-020-01474-8 (PMC7495859; doi:10.1186/s13643-020-01474-8)
Supplement: Supplementary file 3 — Additional file 3: Data extraction tables. [file 13643_2020_1474_MOESM3_ESM.docx]

**Additional file 3. Data extraction table**

| Study ID *(author, year)* | Source | Country, Setting | Design | Population *(N, mean age, sex, ethnicity, clinical condition(s), comorbities)* | ACEs exposure *(direct and indirect)*  Assessment of ACEs *(structured interview, validated questionnaire, official records)* | No of ACEs *(1;2;3;4;>4)* | Control group(s)  *(number, mean age, sex, ethnicity)* | Analysis | Types of outcomes^§^ *(scale; unit, method of measurement; length of follow-up):*  1. MSK painful disorder(s)  2. Somatoform painful disorder(s) | Unajusted^¥^ pain / disability; Precision | Ajusted^ᶲ^ pain / disability; Precision | Cumulative effect / Dose response *(Unit)* |
| --- | --- | --- | --- | --- | --- | --- | --- | --- | --- | --- | --- | --- |
|  |  |  |  |  |  |  |  |  |  |  |  |  |

§ Type of outcome: 1. Musculoskeletal (MSK) painful disorders: neck, back, low back, chest wall, upper /lower extremity, temporomandibular joint; 2.Somatoform painful disorders: widespread pain, fibromyalgia, frequent headache, migraine, abdominal pain, non-cardiac pain and pelvic/gynecological pain; ¥ Unajusted pain / disability (RR, OR, HR, IRR, MD, SMD, Cohens f, Hedges’g, F test); Precision (confidence interval (95% CI), P value, SD, IQR, SE) or Raw data: (ACEs, Outcome +); (AECs, Outcome -); (No ACE, Outcome + ); (No AEC,outcomes); ᶲ Ajusted pain (RR, OR, HR, IRR, MD, SMD, Cohens f, Hedges’g, F test); Precision (confidence interval (CI), P value, SD, IQR, SE).
